# Human adult neurogenesis loss corresponds with cognitive decline during epilepsy progression

**DOI:** 10.1016/j.stem.2024.11.002

**Published:** 2024-12-05

**Authors:** Aswathy Ammothumkandy, Luis Corona, Kristine Ravina, Victoria Wolseley, Jeremy Nelson, Nadiya Atai, Aidin Abedi, Nora Jimenez, Michelle Armacost, Lina M. D’Orazio, Virginia Zuverza-Chavarria, Alisha Cayce, Carol McCleary, George Nune, Laura Kalayjian, Darrin J. Lee, Brian Lee, Robert H. Chow, Christianne Heck, Jonathan J. Russin, Charles Y. Liu, Jason A.D. Smith, Michael A. Bonaguidi

**Affiliations:** 1 Department of Stem Cell Biology and Regenerative Medicine, Eli and Edythe Broad Center for Regenerative Medicine and Stem Cell Research, Keck School of Medicine, University of Southern California, Los Angeles, CA 90033, USA; 2 Neurorestoration Center, Keck School of Medicine, University of Southern California, Los Angeles, CA 90033, USA; 3 Department of Physiology & Neuroscience, Zilkha Neurogenetic Institute, Keck School of Medicine, University of Southern California, Los Angeles, CA 90033, USA; 4 Los Angeles General Medical Center, Los Angeles, CA 90033, USA; 5 Department of Neurology, Keck School of Medicine, University of Southern California, Los Angeles, CA 90033, USA; 6 Department of Neurology, Rancho Los Amigos National Rehabilitation Center, Downey, CA 90242, USA; 7 Department of Psychology, Rancho Los Amigos National Rehabilitation Center, Downey, CA 90242, USA; 8 Department of Neurological Surgery, Keck School of Medicine, University of Southern California, Los Angeles, CA 90033, USA; 9 Department of Biomedical Engineering, Viterbi School of Engineering, University of Southern California, Los Angeles, CA 90089, USA; 10 Zilkha Neurogenetic Institute, Keck School of Medicine, University of Southern California, Los Angeles, CA 90033, USA; 11 Department of Neurology, Medical University of South Carolina, Charleston, SC 29425, USA; 12 Department of Biochemistry and Molecular Medicine, University of Southern California, Los Angeles, CA 90033, USA; 13 Department of Gerontology, University of Southern California, Los Angeles, CA 90089, USA; 14 These authors contributed equally; 15 Lead contact

## Abstract

Mesial temporal lobe epilepsy (MTLE) is a syndromic disorder presenting with seizures and cognitive comorbidities. Although seizure etiology is increasingly understood, the pathophysiological mechanisms contributing to cognitive decline and epilepsy progression remain less recognized. We have previously shown that adult hippocampal neurogenesis dramatically declines in MTLE patients with increased disease duration. Here, we investigate when multiple cognitive domains become affected during epilepsy progression and how human neurogenesis levels contribute to it. We find that intelligence, verbal learning, and memory decline at a critical period of 20 years disease duration. In contrast to rodents, the number of human immature neurons positively associates with auditory verbal, rather than visuospatial, learning and memory. Moreover, this association does not apply to mature granule neurons. Our study provides cellular evidence of how adult neurogenesis corresponds with human cognition and signifies an opportunity to advance regenerative medicine for patients with MTLE and other cognitive disorders.

## In brief

Ammothumkandy et al. provide a functional role for adult neurogenesis in human cognition. They show that immature neuron levels benefit verbal learning in mesial temporal lobe epilepsy (MTLE) patients. The decline and eventual loss of immature neurons in MTLE hippocampus occurs during a critical period of disease progression.

## Graphical Abstract

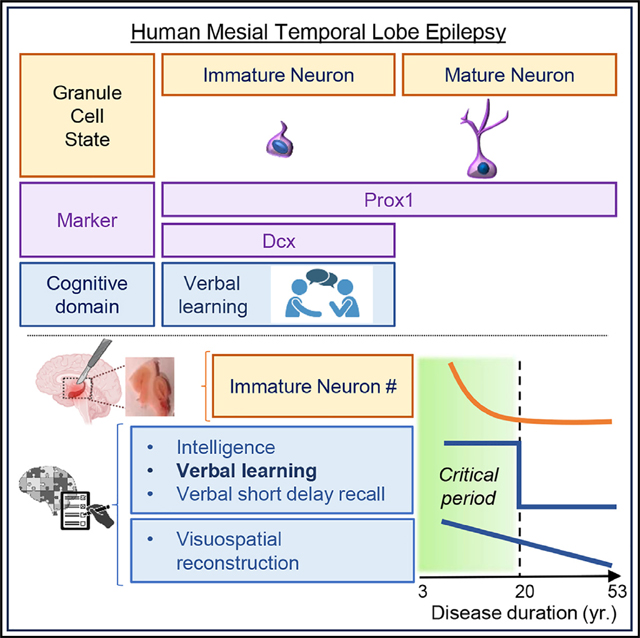

## INTRODUCTION

Epilepsy is a prevalent neurological disorder defined by recurrent seizures and presents comorbidities including neurocognitive dysfunction. Mesial temporal lobe epilepsy (MTLE) is the most common and refractory adult epilepsy, with 70% of patients becoming pharmacoresistant.^1^ In comparison with other epilepsies, MTLE patients are more likely to exhibit disease progression, including worsening seizure control, structural abnormalities, cognitive decline, and behavior impairment.^2^ Cognitive phenotypes in MTLE have been categorized according to clinical criteria and data-driven cluster analyses.^3^ Approximately half of the patients possess intact cognition, while roughly one quarter display impairments in learning, memory, and language, and another quarter display more widespread cognitive dysfunction, including impaired attention, executive function, and processing speed.^3–5^ However, when and which structural abnormalities contribute to cognitive impairment in MTLE patients are not well understood.

MTLE is characterized by focal seizures in the hippocampus, which performs episodic learning and memory, spatial cognition, and emotional processing functions.^6,7^ The human hippocampus also contains a rare niche in which adult neurogenesis can persist throughout life.^8–15^ In this process, neural stem cells produce proliferating neuronal progenitors that differentiate and mature into granule neurons. Functionally, adult immature neurons benefit visuospatial cognition and impede memory interference in rodent models of aging and dementia.^16–19^ However, their role in human cognition remains unknown. A mild decline in hippocampal neurogenesis occurs in healthy people with age,^11^ which is further exacerbated in mild cognitive impairment (MCI) and Alzheimer’s disease (AD).^12,13^ Our recent MTLE study^20^ shows the most profound decline in neurogenesis among patients with neurological diseases to date.^11,13,21^ Neurogenesis rapidly deteriorates over the course of the disease, and immature neurons were not detected after 20 years of MTLE disease duration (DD).^20^ This dynamic range provides an opportunity to define the functional role of neurogenesis in human cognition.

Prior studies indicate that MTLE patients with severe cognitive decline have longer mean DD and implicate that cognitive impairment could be progressive.^3,22,23^ Meanwhile, cognitive decline may be established earlier in disease and remain stable thereafter.^24–26^ Here, we examine a cohort of Spanish-speaking MTLE patients to identify which cognitive domains are affected by DD and when they significantly decline. Sequential neuropsychological and histological analysis in the same MTLE patients reveal which cognitive domains are associated with the number of immature and mature dentate granule neurons. Our results link the decline and eventual loss of immature neurons to verbal learning impairment during a critical period of epilepsy progression.

## RESULTS

### Cognitive performance declines with advancing DD

**Figure 1.**
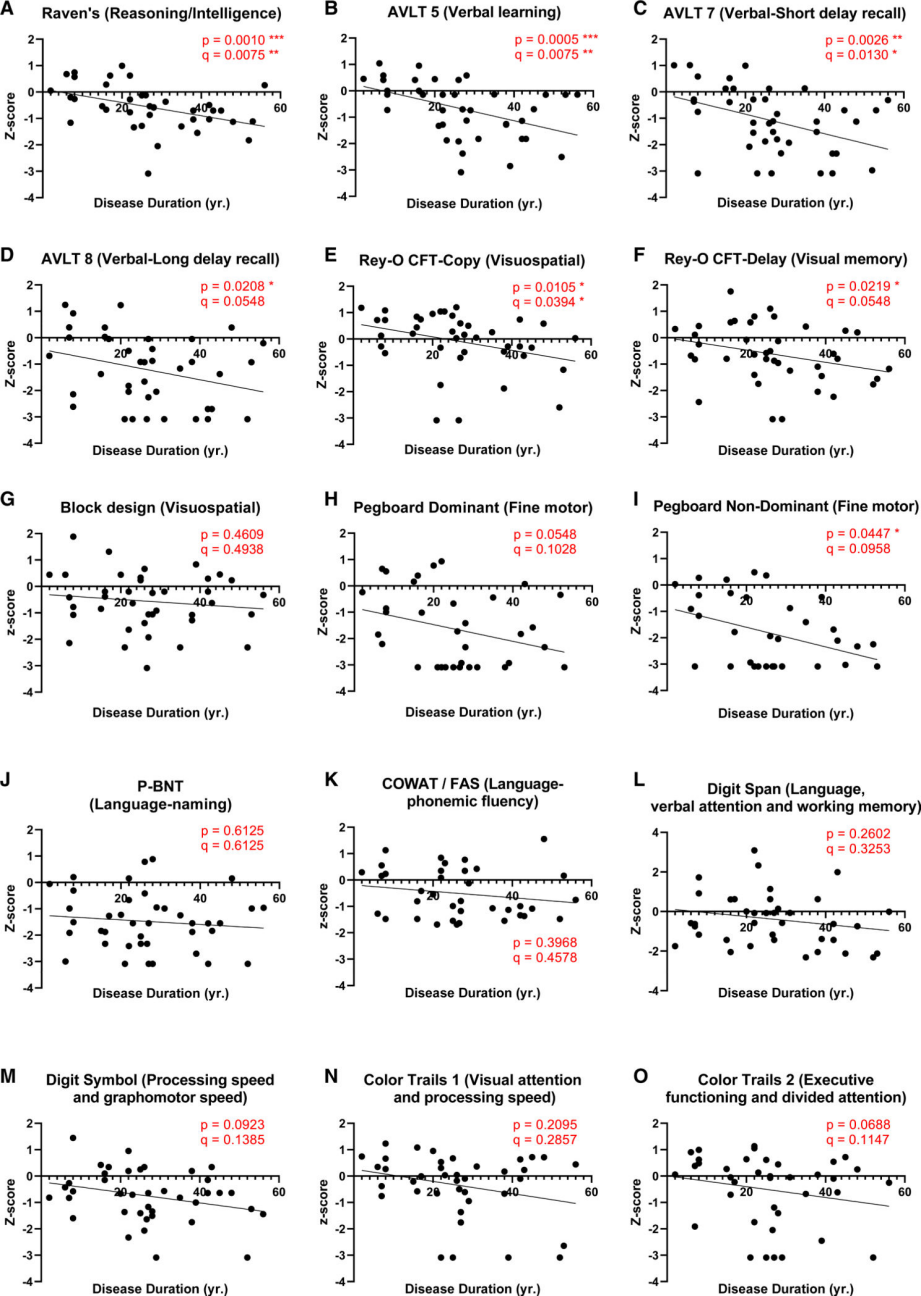
Cognitive performance declines with advancing DD Association between DD and cognitive *Z* scores calculated by two-tailed Spearman’s correlation for 15 cognitive domains, followed by multiple test adjustment using BH-FDR test: (A) Raven’s intelligence, (B) AVLT 5 verbal learning, (C) AVLT 7 verbal short-delay recall, (D) AVLT 8 long-delay recall, (E) Rey-O-CFT copy visuospatial, (F) Rey-O-CFT delay visuospatial memory, (G) block design visuospatial, (H) pegboard dominant fine motor control, (I) pegboard non-dominant fine motor control, (J) P-BNT language, (K) COWAT/FAS language fluency, (L) digit span language attention, (M) digit symbol processing speed, (N) color trails 1 visual attention, and (O) color trails 2 executive functioning. *p* values represent Spearman’s correlation, q values contain BH-FDR correction for 15 cognitive tests, * *p*,q ≤ 0.05, ** *p*, q ≤ 0.01, *** *p*, q ≤ 0.001. See also Table S2.

Cognitive functioning was evaluated pre-surgically using the Neuropsychological Screening Battery for Hispanics (NeSBHIS).^27,28^ We first identified which cognitive domains from the NeSBHIS (Table 1) were affected by DD. Education-normalized NeSBHIS *Z* scores and age plus education-normalized *Z* scores for grooved pegboard fine motor test^27^ were analyzed from MTLE patients (*N* = 40), with average age (M = 39.88 years; SD = 11.56), DD (M = 26.74 years; SD = 14.05), and reported years of education (M = 7.9 years; SD = 3.51) (Table S1). Spearman’s correlation followed by multiple test adjustment using Benjamini and Hochberg original false discovery rate (BH-FDR) revealed declining performances across multiple cognitive domains (Figure 1; Table S2). Hippocampal-dependent functions of verbal learning and memory^29^ significantly declined on learning (Figure 1B) and short-delay recall trials (Figure 1C) tests. The verbal long-delay recall trial (Figure 1D) also significantly declined with DD, as shown by Spearman’s test; however, it did not withstand multiple corrections with BH-FDR. In addition to verbal domains, reasoning and intelligence (Figure 1A)^22^ and visuospatial skills (Figure 1E) showed significant declines with DD, withstanding BH-FDR corrections. Meanwhile, visual memory (Figure 1F) and fine motor non-dominant hand (Figure 1I) tests showed significant decline with DD, but did not withstand BH-FDR corrections. The remaining cognitive domains, including language, attention, processing speed, and executive functioning, did not show significant associations with DD (Figure 1). Hence, this cohort of Spanish-speaking MTLE patients present progressive cognitive decline with DD in intelligence and both verbal and visuospatial domains.

### A critical period of cognitive impairment in MTLE patients

**Figure 2.**
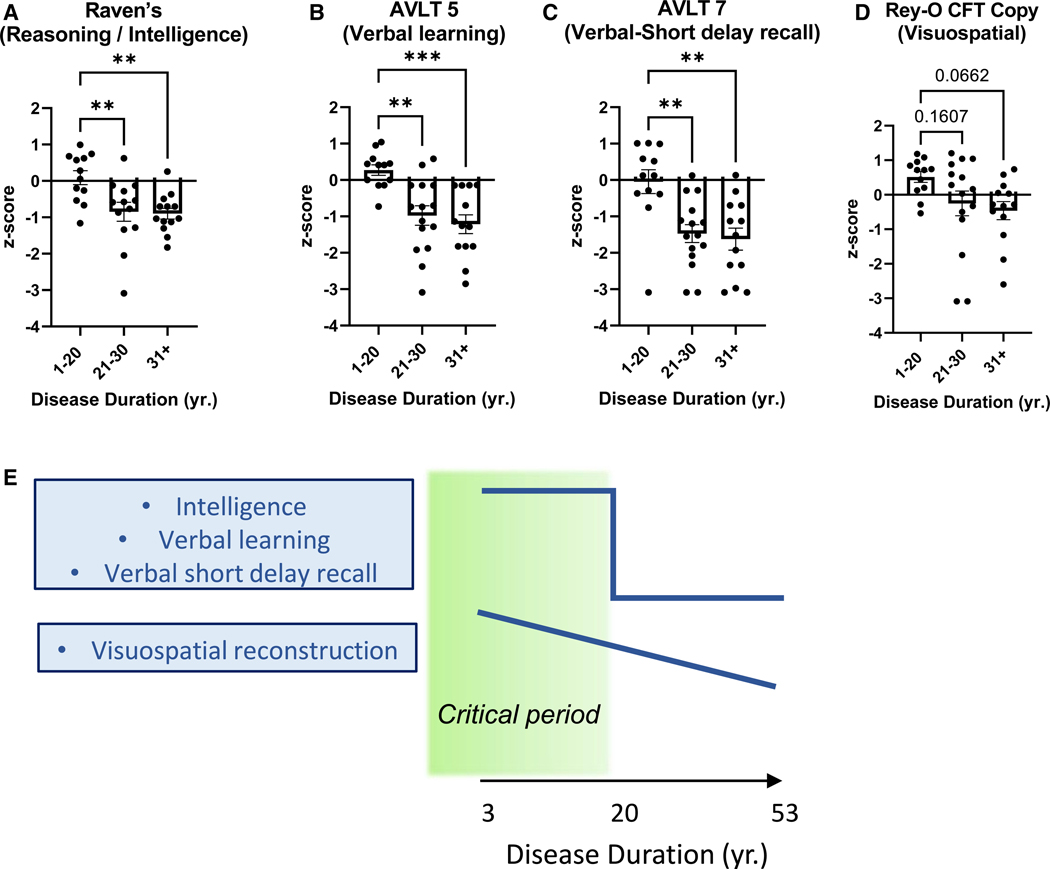
A critical period of cognitive impairment in MTLE patients (A–C) Three DD groups were created; G1: 1–20 years DD, G2: 21–30 years DD, and G3: 31+ years DD. Cognitive *Z* scores among 3 groups were compared using a one-way ANOVA with post hoc Tukey’s analysis: (A–C) cognitive *Z* scores are significantly higher for G1 compared with G2 and G3: (A) Raven’s intelligence, (B) AVLT 5 verbal learning, and (C) AVLT 7 verbal short-delay recall. (D) Cognitive *Z* scores are not significantly different between groups for Rey-O CFT copy visuospatial test. * *p* ≤ 0.05, ** *p* ≤ 0.01, *** *p* ≤ 0.001. All error bars represent SEM across individual cases. (E) Intelligence, verbal learning, and verbal short-delay recall decline at an early critical period, whereas visuospatial skill declines progressively. See also Figure S1 and Table S3.

To identify the critical period when cognitive performance significantly decreases, we separated the patient data into three similarly powered distinct groups based on DD. Group 1 (G1, *N* = 12) was < 20 years DD, group 2 (G2, *N* = 15) was 21 to 30 years DD, and group 3 (G3, *N* = 13) contained > 31 years DD. We first compared whether the NeSBHIS *Z* scores differed significantly between groups (Table S3). We then focused analysis on the cognitive tests that exhibited a pronounced negative association with DD after multiple corrections (Figures 1A–1C and 1E). For intelligence (Figure 2A), verbal learning (Figure 2B), and verbal short-delay recall memory (Figure 2C), G2 and G3 had significantly lower *Z* scores compared with G1; meanwhile, scores did not decline significantly from G2 to G3. This result indicates a sudden drop in performance at an earlier critical period for intelligence and verbal domains. Meanwhile, visuospatial skills did not decline between G1, G2, and G3 (Figure 2D) despite the significant gradual decline observed with DD (Figure 1E). In addition, some tests that did not show a multiple corrections adjusted decline in DD (Figure 1) displayed a significant drop in performance between G1 and G2, including verbal long-delay recall (Figure S1D) and fine motor test pegboard for dominant hand (Figure S1H) and non-dominant hand (Figure S1I). Lower *Z* scores for the remaining tests did not display significant differences between any DD groups (Figure S1). Therefore, the most significant declines in intelligence, verbal learning, and memory, but not visuospatial skills, occur in MTLE patients before a critical period of 20 years (Figure 2E), revealing a window for therapeutic interventions to reduce the impact of specific cognitive comorbidities.

### Contributions of immature and mature granule neurons to cognition

**Figure 3.**
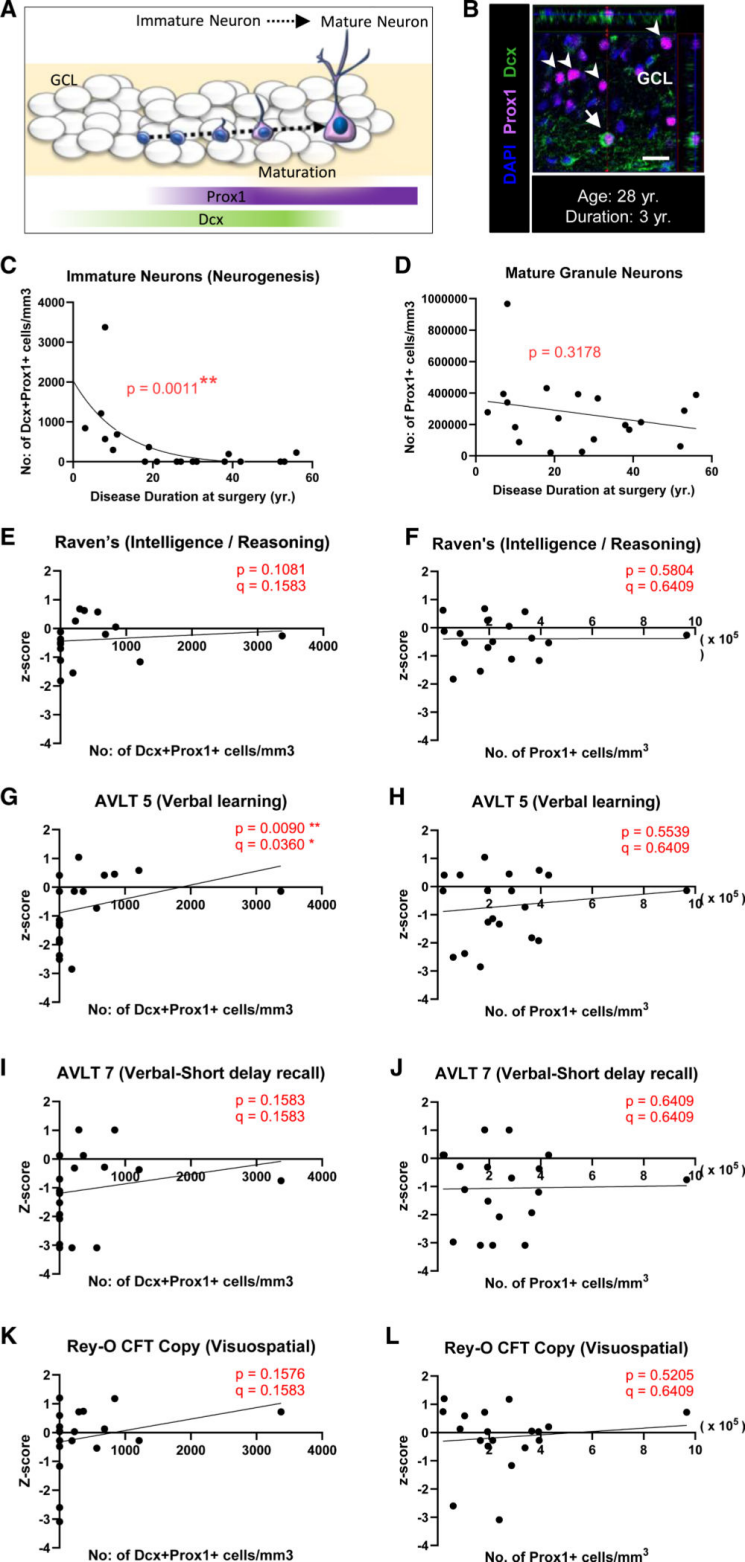
Contributions of immature and mature granule neurons to cognition (A) Cartoon illustration of Dcx and Prox1 expression across stages of granule neuron maturation. (B) Dcx+ (green) Prox1+ (purple) immature neurons (arrow) and Dcx-Prox1+ mature granule neurons (arrowhead) identified in the granular cell layer (GCL) of adult human MTLE cases. Scale bar represents 20 μm. (C) Number of Dcx+ Prox1+ immature neurons/mm^3^ in the GCL with DD (yr.) at surgery. Two-tailed Spearman’s correlation ** *p* ≤ 0.0011. Spearman’s r = −0.6899. (D) Number of Prox1+ granule neurons/mm^3^ in the GCL with DD (yr.) at surgery. Two-tailed Spearman’s correlation *p* = 0.3178 Spearman’s r = −0.2422. (E, G, I, and K) Two-tailed Spearman’s correlation between the number of Dcx+ Prox1+ immature neurons/mm^3^ detected from the surgically resected hippocampus and presurgical cognitive *Z* scores for (E) Raven’s intelligence, (G) AVLT 5 verbal learning, (I) AVLT 7 verbal short-delay recall, and (K) Rey-O CFT Copy visuospatial test. (F, H, J, and L) Two-tailed Spearman’s correlation between number of Prox1+ granule neurons/mm^3^ detected from the surgically resected hippocampus and presurgical cognitive *Z* scores for (F) Raven’s intelligence, (H) AVLT 5 verbal learning, (J) AVLT 7 verbal short-delay recall, and (L) Rey-O CFT copy visuospatial test. *p* values represent Spearman’s correlation, q values correct using a hypothesis-based nested BH-FDR test for 4 cognitive tests, * *p*, q ≤ 0.05, ** *p*, q ≤ 0.01, *** *p*, q ≤ 0.001. See also Figures S2, S3, and Table S4.

The identified critical period (Figure 2) coincides with the disease progression when immature neurons are reported to become nearly undetectable in MTLE patients.^20^ We therefore performed a hypothesis-based investigation into the relationship between immature neuron levels and performance on cognitive tests that exhibit significant decline with DD and withstand multiple corrections testing—including intelligence, verbal learning, verbal short-delay recall, and visuospatial tests (Figures 1A–C and 1E). We first benchmarked our previous observation of immature neuron decline with DD to our current cohort of Spanish-speaking patients only (the current cohort contains a subset of patients from our previous study plus 6 additional cases). Immature neurons were quantified as the number of Dcx+Prox1+ cells (Figures 3A and 3B) by performing histology in surgically resected tissue (*N* = 19). Consistent with our previous report,^20^ we observed an exponential decline of adult immature neuron numbers with DD (Figure 3C). Next, we investigated the association of immature granule neuron levels with (1) intelligence, (2) verbal learning, (3) verbal short-delay recall, and (4) visuospatial tests using a Spearman’s correlation test followed by a nested BH-FDR correction for these 4 tests (Table S4). We observed that verbal learning showed a significant positive association with immature neuron levels (Figure 3G). To further unravel whether this association is enriched to immature granule neurons, we investigated the contribution of mature granule neurons. The number of mature granule neurons (Prox1+ cells) observed no significant associations with DD (Figure 3D) nor verbal learning (Figure 3H). No associations among immature nor mature neurons were observed with intelligence, verbal short-delay recall, and visuospatial tests (Figures 3E, 3F, and 3I–3L). Therefore, immature rather than mature granule neuron levels particularly benefit verbal learning. Further, we explored the relationship between immature and mature granule neurons with all 15 cognitive tests, regardless of their association with DD (Figures S2 and S3; Table S4). Immature neurons showed a significant positive association with verbal learning, verbal long-delay recall, and language (phonemic fluency), whereas mature granule neurons showed significant positive correlation only with language (phonemic fluency). However, none of these associations were strong enough to withstand BH-FDR correction for 15 tests (Figures S2 and S3; Table S4). Hence, our study identifies that immature and mature granule neuron numbers can be associated with distinct cognitive functions at varying strengths, with the strongest association observed between immature neurons and verbal learning (Figure 3I). The study provides cellular evidence for an association between immature neurons and specific cognitive domains in humans.

## DISCUSSION

In MTLE, when and which structural abnormalities contribute to the progression of cognitive impairment are not well understood. Here, we find that human adult immature neuron loss corresponds with auditory verbal learning (AVLT 5) impairment during a critical DD of 20 years. Although we observed a similarly pronounced initial decline in intelligence (Raven’s) and verbal memory (AVLT 7), plus a gradual decline in visuospatial skills (Rey-O Copy), these domains are less associated with immature neuron levels. We also detected a weak positive association between immature neurons and verbal long-delay recall (AVLT 8), whereas both immature and mature granule neuron states show a weak positive association with language fluency (FAS). These observations illustrate an interplay between neurogenesis levels and the timing of cognitive decline, potentially across multiple domains in MTLE patients.

The role of immature neurons in specific human cognitive functions has remained unknown. A prior study of MTLE patients related lower proliferation of neural progenitors *in vitro* to impaired memory on the intracarotid sodium amobarbital test.^30^ More recently, findings in patients with MCI identified that proliferating neuroblast numbers benefit global cognition.^12^ Our analysis of multiple cognitive domains was critical in understanding which neuropsychological functions are influenced by adult human neurogenesis. Remarkably, finding that levels of immature neurons, more so than mature granule neurons, particularly benefit verbal learning in MTLE patients alludes to a specialized function of adult neurogenesis. In rodents, newborn granule cells possess enhanced intrinsic excitability, stronger responsiveness to external inputs,^31^ and enhanced synaptic plasticity,^7,32,33^ which contributes to learning, working memory, and memory consolidation. Whether these properties are evolutionarily conserved in human new-born granule neurons remains to be tested. Meanwhile, mature granule neurons facilitate memory recall,^8^ which we also observe via the FAS language test that involves rapid retrieval of long-term crystalized, verbal knowledge of language, or semantic memory. The shared weak benefit of immature and mature neurons to outcomes on the FAS language test might reflect cumulative granule neuron turnover—neuronal cell loss and cell genesis—during human lifespan.^8^ Thus, the current neurogenesis findings provide a different understanding of the cellular dynamics that link dentate gyrus volume change to cognitive outcomes.^29,34^

Learning and memory impairment reflect hippocampal network abnormalities that extend beyond epilepsy to other neurodegenerative disorders.^6,35^ Learning and working memory diminish as individuals age.^36^ Further, both MCI and AD are associated with impairments in the acquisition, consolidation, and retrieval of verbal information, with AD patients displaying more severe deficits.^37–39^ Reduced verbal learning ability over repeated exposures also represents a key neuropsychological marker of AD that predicts functional decline.^38,39^ As hippocampal neurogenesis deficits have been reported in patients at early stages of both MCI and AD,^12,13^ immature neuron levels may more broadly contribute to verbal learning and memory across neurological conditions. This study reveals impacted cognitive domains that are shared (language) or distinct between immature and mature adult granule neurons. These findings serve as a crucial basis for identifying potential clinical interventions aimed at preventing or reversing cognitive decline in MTLE and other cognitive disorders. For example, a previous study in healthy individuals has also implied a role for exercise-induced neurogenesis in verbal learning using indirect measures of dentate gyrus cerebral blood volume.^40^ Our more direct assay, quantifying the amount of immature and mature granule neurons in hippocampal resections from MTLE patients, indicates that neurogenesis benefits multiple cognitive domains, with the strongest association for verbal learning. Further research is needed to understand whether therapeutic or lifestyle changes that promote neurogenesis and cognition in model systems can be as regenerative in humans.

### Limitations of the study

The levels of immature or mature granule neurons are likely not the only parameters to affect altered cognitive performance in MTLE patients. This study’s small size limits our ability to detect less-dramatic cell type associations with cognitive domains. We therefore interpret significant (*p* < 0.05) cellular phenotypes that do not satisfy multiple test corrections as weak associations. We are also insufficiently powered to analyze the contribution of seizure burden, anti-epilepsy medications, etiological risk factors, and sex to neurogenesis levels, verbal and visual learning and memory, and their lateralization between left and right hippocampus.^41^ However, early childhood onset (<5 years) does not appear to impact verbal learning and memory in this patient cohort (Figure S4).^25^ Finally, we do not conclude that neurogenesis could lack contribution to visuospatial learning and memory in people, as has been related in rodent studies.^18^ Such species divergence could reflect our study not assessing repeated visual learning and encoding. Future studies may investigate visuospatial learning using encoding tests such as the Brief Visuospatial Memory Test-Revised (BVMT-R), which requires learning a series of figures presented over repeated trials^42^ or visual recognition memory performance (pattern separation) using the mnemonic similarity test (MST).^43,44^

### RESOURCE AVAILABILITY

#### Lead contact

Further information and requests for resources and reagents should be directed to and will be fulfilled by the lead contact, Michael A. Bonaguidi (mbonagui@usc.edu).

#### Materials availability

The availability of human MTLE surgically resected hippocampal tissue is limited. Any information regarding the availability of materials can be directed to M.A.B.

#### Data and code availability

- De-identified patient data have been deposited at Mendeley Data. They are publicly available as of the date of publication. Accession numbers are listed in the key resources table. Any information regarding the availability of raw data can be directed to the lead contact, M.A.B.
- This paper does not report original code.
- Any additional information required to reanalyze the data reported in this paper is available from the lead contact, M.A.B., upon request.

### EXPERIMENTAL MODEL AND STUDY PARTICIPANT DETAILS

#### Human participants

The cognitive study was approved by University of Southern California Institutional Review Board approval number: HS-10–00162, and Rancho Los Amigos National Rehabilitation Center and the Rancho Research Institute’s Institutional (RRI) Review Board, approval number: RRI IRB # 195 and the data use agreement with RRI and the University of Texas Southwestern Medical Center IRB Protocol approval number: STU-052018–086. We have complied with all relevant ethical regulations. Written consents were obtained from the patients to study the clinical and demographic information. De-identified patient clinical details (age and DD of epilepsy at the time of Neuropsychological test, age of disease onset, sex, left vs. right hemisphere of affected hippocampus, education years, ethnicity, nationality) are provided in Table S1. Mesial temporal lobe sclerosis (MTS) was determined by magnetic resonance imaging and confirmed by pathology reports to be present in N = 38/39 patients. Both male (N = 16) and female (N = 24) cases were included to examine sex as a biological variable in the cognitive analysis performed in the study. Sex based analyses was not performed due to limited sample size. We also do not have data available on ancestry, and socioeconomic status. These are limitations to our research’s generalizability.

For the study of surgically resected epilepsy tissue, written consents for the surgical treatment of MTLE, tissue donation for the study as well as relevant clinical and demographic information collection (University of Southern California Institutional Review Board approval number: HS-17–00370) were obtained from all the patients prior to the surgery. Human hippocampal tissue was obtained from 19 out of 40 patients undergoing an *en bloc* resection of their hippocampus for the treatment of medically resistant MTLE, following strict ethical guidelines. Both male (N = 8) and female (N = 11) cases were included to examine sex as a biological variable in the study. Sex based analyses was not performed due to limited sample size and therefore is a limitation to our research’s generalizability.

### METHOD DETAILS

#### Cognitive assessment

Retrospective cognitive analysis of presurgical neuropsychological exams of Spanish-speaking patients (N=40) with intractable TLE was performed to explore the relationship between DD and TLE with cognitive functioning. The Neuropsychological Screening Battery for Hispanics (NeSBHIS) was part of a comprehensive presurgical evaluation. Intellect, attention, processing speed, language, visuospatial, visual memory, verbal learning and memory, executive functioning and fine motor dexterity were measured to assess epilepsy comorbidities (Table S1). To account for variability in education, which influences neuropsychological test performance,^27^ subtest raw scores were converted to z-scores using education-based normative data.^28^ Grooved pegboard was used instead of the fine-motor Pin test from NeSBHIS, which was normalized using age and education based normative data as in Smith et al..^27^ Upper and lower cut off values for z scores were set at +/− 3.09 to eliminate potential outliers due to very superior performance (z-scores > +3.09) and clinically profound impairment (<3.09).

#### Human MTLE Hippocampal Tissue processing

The surgery was performed by neurosurgeons at the Keck Medical Center or Los Angeles General Medical Center. The surgery was performed within a year of neuropsychology test for 10 out of 19 patients. The rest of the cases underwent surgery within 5 years of test (N = 4 after 1 year, N = 1 after 2 years, N = 2 after 3 years, N = 2 after 4 years). DD at surgery is provided in Extended Data Table 2. Surgically resected hippocampal tissue was transported to the laboratory within 30 minutes to 1 hour of surgical removal in a tube containing HypoThermosol^®^ FRS (BioLife Solutions, Bothell, WA) solution maintained at approximately 4˚C in an ice box.

The storage and method of sectioning human surgical specimens, type of fixative, and duration in fixative are important factors when performing immunocytochemistry for adult neurogenesis studies.^13^ Hippocampal tissue was cut into 400 μm thick slices in chilled (4˚C), oxygenated (95% O_2_, 5% CO_2_) N-methyl-D-glycamine - artificial cerebrospinal fluid (aCSF) solution (NMDG-aCSF; NMDG 93mM; KCl 2.5mM; NaH_2_PO_4_ 1.2mM; NaHCO_3_ 30mM; HEPES 20mM; Glucose 25mM; Sodium ascorbate 5mM; Thiourea 2mM; Sodium pyruvate 3mM; MgSO_4_ 10mM; CaCl_2_ 0.5mM) using a vibrating microtome (Leica VT1200S, Leica Biosystems, Germany). Vertical deflection of the blade was minimized with Vibrocheck technology (Leica Biosystems, Germany). Slicing parameters were set at speed 0.15–0.5 mm/s and vibration amplitude 1.5 mm. Hippocampal slices were first used for multi electrode array experiments explained in our previous study.^20^ Slices were allowed to recover for a minimum of 1 hour in a chamber with oxygenated (95% O_2_, 5% CO_2,_ 32˚C) aCSF using one of the following formulations (Formulation 1: NaCl 124 mM; KCl 4 mM; NaHCO_3_ 26 mM; Glucose 10 mM; CaCl_2_ 2mM; MgCl_2_ 2mM solution, Formulation 2: NaCl 124 mM; KCl 2.5 mM; NaH_2_PO_4_ 1.2 mM, NaHCO_3_ 24 mM; HEPES 5mM, Glucose 12.5 mM; CaCl_2_.2H_2_O 2mM; MgSO_4_.7H_2_O 2mM) before multi electrode array (MEA) recording. Continuous oxygenated, warm (32^◦^C) aCSF at a rate of 6 ml/min was circulated in the MEA ring well during tissue recording. A 5 min baseline recording was performed in aCSF followed by a 20–30 min recording in a modified aCSF solution containing high (8 mM) potassium, low (0.25 mM) magnesium and 100 μM 4-aminopyridine (4-AP) to induce inter-ictal like activity. Detailed MEA method is explained in our previous study identifying neurogenesis in human MTLE.^20^ Following the MEA recording, slices were immediately fixed using 4% PFA for immunohistochemistry.

#### Immunohistochemistry and cell quantification

Slices were pre-treated in a similar manner for methodological consistency between cases. Slices were fixed in 4% PFA - made freshly from 16% PFA stored at −20^◦^C - for 30 minutes at room temperature and washed thoroughly with 1x phosphate buffered saline (PBS) overnight. The slices were dehydrated using 30% sucrose in PBS at 4 ^◦^C. After 1 week, once the slice sank it was embedded in O.C.T compound (Tissue-Tek), ensuring the DG region is flat. Slices were further sub-sectioned by cryostat (Leica CM 3050 S) into 30mm and mounted to super frost plus slides. The slides were dried overnight at room temperature and transferred to −20^◦^C for long term storage. Before performing immunohistochemistry, slides were equilibrated to room temperature for 2 hours or at 37^◦^C for 30 minutes. Slides were further washed in PBS for 1 hour, followed by blocking and permeabilization in 3% Bovine serum albumin (BSA), 10% Donkey serum in 0.1% Triton-X in PBS for 2 hours at room temperature. The sections were incubated with primary antibodies for 18–20 hours at 4^◦^C in 0.3 ≤ BSA, 1% Donkey serum and 0.1% Triton X in PBS. Following a 1-hour equilibration to room temperature, slides were washed in 0.1% Triton X in PBS for 15 minutes 3 times. Secondary antibodies from Jackson Immuno research laboratories were diluted from a stock solution of 0.6mg/ml and incubated along with 1μg/ml of DAPI (Roche, Cat No: 10236276001) for 2 hours at room temperature in 0.3 ≤ BSA, 1% Donkey serum and 0.1% Triton X. Sections were washed 3 times in 0.1% Triton X in PBS for 15 minutes and mounted with mounting media prepared in the lab. Glycerol-based PVA −2.5% DABCO mounting media was prepared by first adding 4.8 g PVA (Sigma P8136) to 12 g glycerol, mixing well, and adding 12 ml distilled water. After overnight mixing in rotor, 24 ml 0.2M Tris-HCL pH 8–8.5 was added, and the tube was heated to 50^◦^C for 10–30 minutes in water bath with periodic mixing until the mixture was completely dissolved. The tube was further centrifuged at 5000g for 15 minutes and the supernatant was collected. 1.25 g DABCO (Sigma D2522) was added to 50 ml supernatant, heated to 50^◦^C for 10–30 minutes and then centrifuged at 5000g for 15 min to collect the supernatant. The supernatant mounting media mix was aliquoted and stored at −20^◦^C for long term storage and thawed at room temperature for mounting the tissue. After mounting media solidifies between the slide and cover glass whole DG region was imaged at 20X using a Zeiss LSM 700 confocal microscope automated tile scan. All cell quantification was performed manually in Zeiss blue software. GCL boundaries were marked using the DAPI channel around the seemingly compact cell layer leaving a gap of two cell nuclear distance on both sides of the hilus and molecular layer. Each whole section was analysed to quantify the number of rare Dcx+ Prox1+ cells. In addition to newborn neurons, Dcx is expressed by astroglia (Dcx+ Prox1- S100B+) which are abundantly present in MTLE tissue, but not healthy subjects. The abundance of Dcx+ cells in MTLE patients provides an internal control to assay overall staining quality on each specific specimen. Dcx staining is extremely sensitive to tissue health, unlike Prox1 staining whose intersection with Dcx denotes the presence of immature neurons. Indeed, some specimens (5 of 24 Spanish-speaking patients) were excluded from the study for broadly lacking Dcx expression - potentially due to poor tissue health (Ammothumkandy et al., Nature Neuroscience 2022- Figure 2^,20^). For the quantification of mature Prox1+ granule neurons, representative sub regions in the GCL were analysed due to their high prevalence. The remaining statistical analysis and plots were prepared using Prism10 from GraphPad Inc.

**Table:** 

Primary Antibodies				

Antibodies	Species	Dilution	Company	Catalog No:
Doublecortin (Dcx)	Mouse	1 in 33	Santa Cruz	sc-390645
Prox1	Rabbit	1 in 200	Abcam	ab101851

Secondary Antibodies				

Antibodies	Species	Dilution	Company	Catalog No:
Anti-mouse Alexa Fluor 488	Donkey	1 in 200	Jackson Immunoresearch	715–546-151
Anti-rabbit Alexa Fluor 488	Donkey	1 in 200	Jackson Immunoresearch	711–546-152
Anti-mouse Cy3	Donkey	1 in 200	Jackson Immunoresearch	715–166-150
Anti-rabbit Cy3	Donkey	1 in 200	Jackson Immunoresearch	711–166-152
Anti-rabbit Alexa Fluor 647	Donkey	1 in 200	Jackson Immunoresearch	711-606-152

#### QUANTIFICATION AND STATISTICAL ANALYSIS

All the statistical analysis was performed using GraphPad Prism 10. The statistical methods used for each Figure is specifically noted in the Figure legends. N value (number of patients) for each individual analysis is noted in the data tables (Tables S3–S6). No statistical methods were used to predetermine sample size. No data points were excluded when available. Investigators were blinded to experimental parameters and group allocations. Data distribution was assumed to be normal, but this assumption was not formally tested. Individual data points are shown in all the graphs. A two-tailed Spearman’s correlation was performed to calculate the strength of linear relationship between two variables in Figures 1 and 3. This analysis was followed by a multiple test adjustment for the number of correlation tests compared using Benjamini and Hochberg Original False Discovery Rate (BH-FDR) method. The N value, correlation coefficient, p-value and q-value for these analyses are reported in Tables S3, S5, and S6). To identify the critical window of cognitive decline in Figure 2, the cohort was divided into three similarly powered DD groups; < 20 years (G1, N = 12), 21 to 30 years (G2, N =15), and > 31-years (G3, N =13), allowing ANOVA analyses, followed by a Tukey’s multiple comparison post-hoc test. The F statistic, p-value, and Tukey’s multiple comparison test results are reported in Table S4. A comparison of fits among linear regression, logarithmic, and exponential one phase decay was performed for Figure 3C. The parameters for goodness of fit were slightly better (R^2^ value closer to 1, lower RMSE) for exponential compared to logarithmic and linear regressions.

**Table:** 

	R^2^	RMSE

Linear	0.262	686.4
Exponential one phase decay	0.3852	626.5
Logarithmic	0.3819	628.1

## Supplementary Material

1

## Figures and Tables

**Table 1.** Cognitive domains and the respective NeSBHIS tests administered to MTLE patients, related to all figures and tables

Cognitive domain	Test (description)

Reasoning/intelligence	Raven’s standard progressive matrices (nonverbal/visual reasoning task to estimate general intelligence; often regarded as culture fair measurement)
Attention, processing, speed, and executive functioning	digit symbol (processing speed and graphomotor speed) color trails 1 (visual attention and processing speed) color trail 2 (divided attention, cognitive flexibility, and executive functioning)
Language/verbal ability	Pontón-Satz Boston naming test (P-S BNT; confrontational object naming drawn figures) controlled oral word association test (COWAT, FAS; measure of phonemic verbal fluency) digit span (verbal attention and working memory, repetition of digits forward and then reverse)
Verbal learning and memory	World Health Organization – University of California, Los Angeles Auditory Verbal Learning Test (WHO-UCLA AVLT) trials 1–5 (15 words presented over five trials to promote learning) trial 6 (second 15-word list, an interference trial) trial 7 (short-delay recall of the original items, immediately follows trial 6) trial 8 (20-min, long-delay recall)
Visuospatial	block design (visuospatial constructional ability) Rey-Osterrieth complex figure test (ROCFT) – copy (reconstruction of an intricate, two-dimensional geometric figure)
Visual memory	ROCFT – delay (10-min-delayed recall, reconstructing the ROCFT stimuli)
Fine motor functioning	grooved pegboard dominant (GP-D) and non-dominant (GP-ND) hands (task of manual dexterity, visuomotor integration, and speed)

**Table:** KEY RESOURCES TABLE

REAGENT or RESOURCE	SOURCE	IDENTIFIER

Antibodies		

anti-Doublecortin (E-5) Mouse monoclonal	Santa Cruz	Cat#sc-390645; RRID: AB_3626313
anti-Prox1 Rabbit Polyclonal	Abcam	Cat# ab101851; RRID: AB_10712211

Biological samples		

Human Mesial Temporal Lobe Epilepsy (MTLE) surgically resected hippocampus brain tissue	Keck Medical Center and Los Angeles General Medical Center	N/A

Deposited data		

MTLE patient demographics, neuropsychological performance, neurogenesis levels	This paper; Mendeley data	https://www.doi.org/10.17632/t5b79gwt3y.1

Software and algorithms		

Zen black 2012 SPS (Image acquisition)	ZEISS ZEN Microscopy Software	RRID:SCR_018163
Zen 3.1 blue (Image analysis)	ZEISS ZEN Microscopy Software	RRID:SCR_013672
GraphPad Prism 10	Graphpad	RRID:SCR_002798

## Highlights

- MTLE patients present progressive cognitive decline
- Intelligence, verbal learning, and memory decline around 20 years’ disease duration
- This critical period coincides with an exponential loss of immature granule neurons
- Immature granule neuron numbers positively correlate with verbal learning performance

## References

[1] Engel J; International League Against Epilepsy (ILAE) (2001). A proposed diagnostic scheme for people with epileptic seizures and with epilepsy: report of the ILAE Task Force on Classification and Terminology. Epilepsia 42, 796–803. 10.1046/J.1528-1157.2001.10401.X. 11422340

[2] Coan AC, and Cendes F. (2013). Epilepsy as progressive disorders: What is the evidence that can guide our clinical decisions and how can neuroimaging help? Epilepsy Behav. 26, 313–321. 10.1016/J.YEBEH.2012.09.027. 23127969

[3] Hermann B, Seidenberg M, Lee EJ, Chan F, and Rutecki P. (2007). Cognitive phenotypes in temporal lobe epilepsy. J. Int. Neuropsychol. Soc 13, 12–20. 10.1017/S135561770707004X. 17166299

[4] Baxendale S, and Thompson P. (2020). The association of cognitive phenotypes with postoperative outcomes after epilepsy surgery in patients with temporal lobe epilepsy. Epilepsy Behav. 112, 107386. 10.1016/J.YEBEH.2020.107386. 32911298

[5] Elverman KH, Resch ZJ, Quasney EE, Sabsevitz DS, Binder JR, and Swanson SJ (2019). Temporal lobe epilepsy is associated with distinct cognitive phenotypes. Epilepsy Behav. 96, 61–68. 10.1016/j.yebeh.2019.04.015. 31077942

[6] Knierim JJ (2015). The hippocampus. Curr. Biol 25, R1116–R1121. 10.1016/j.cub.2015.10.049. 26654366

[7] Hainmueller T, and Bartos M. (2020). Dentate gyrus circuits for encoding, retrieval and discrimination of episodic memories. Nat. Rev. Neurosci 21, 153–168. 10.1038/S41583-019-0260-Z. 32042144 PMC7115869

[8] Spalding KL, Bergmann O, Alkass K, Bernard S, Salehpour M, Huttner HB, Boström E, Westerlund I, Vial C, Buchholz BA, (2013). Dynamics of Hippocampal Neurogenesis in Adult Humans. Cell 153, 1219–1227. 10.1016/j.cell.2013.05.002. 23746839 PMC4394608

[9] Eriksson PS, Perfilieva E, Björk-Eriksson T, Alborn AM, Nordborg C, Peterson DA, and Gage FH (1998). Neurogenesis in the adult human hippocampus. Nat. Med 4, 1313–1317. 10.1038/3305. 9809557

[10] Knoth R, Singec I, Ditter M, Pantazis G, Capetian P, Meyer RP, Horvat V, Volk B, and Kempermann G. (2010). Murine features of neurogenesis in the human hippocampus across the lifespan from 0 to 100 years. PLoS One 5, e8809. 10.1371/journal.pone.0008809. 20126454 PMC2813284

[11] Boldrini M, Fulmore CA, Tartt AN, Simeon LR, Pavlova I, Poposka V, Rosoklija GB, Stankov A, Arango V, Dwork AJ, (2018). Human Hippocampal Neurogenesis Persists throughout Aging. Cell Stem Cell 22, 589–599.e5. 10.1016/j.stem.2018.03.015. 29625071 PMC5957089

[12] Tobin MK, Musaraca K, Disouky A, Shetti A, Bheri A, Honer WG, Kim N, Dawe RJ, Bennett DA, Arfanakis K, and Lazarov O. (2019). Human Hippocampal Neurogenesis Persists in Aged Adults and Alzheimer’s Disease Patients. Cell Stem Cell 24, 974–982.e3. 10.1016/j.stem.2019.05.003. 31130513 PMC6608595

[13] Moreno-Jiménez EP, Flor-García M, Terreros-Roncal J, Rábano A, Cafini F, Pallas-Bazarra N, Ávila J, and Llorens-Martín M. (2019). Adult hippocampal neurogenesis is abundant in neurologically healthy subjects and drops sharply in patients with Alzheimer’s disease. Nat. Med 25, 554–560. 10.1038/s41591-019-0375-9. 30911133

[14] Wang W, Wang M, Yang M, Zeng B, Qiu W, Ma Q, Jing X, Zhang Q, Wang B, Yin C, (2022). Transcriptome dynamics of hippocampal neurogenesis in macaques across the lifespan and aged humans. Cell Res. 32, 729–743. 10.1038/S41422-022-00678-Y. 35750757 PMC9343414

[15] Zhou Y, Su Y, Li S, Kennedy BC, Zhang DY, Bond AM, Sun Y, Jacob F, Lu L, Hu P, (2022). Molecular landscapes of human hippocampal immature neurons across lifespan. Nature 607, 527–533. 10.1038/s41586-022-04912-w. 35794479 PMC9316413

[16] Horowitz AM, Fan X, Bieri G, Smith LK, Sanchez-Diaz CI, Schroer AB, Gontier G, Casaletto KB, Kramer JH, Williams KE, and Villeda SA (2020). Blood factors transfer beneficial effects of exercise on neurogenesis and cognition to the aged brain. Science 369, 167–173. 10.1126/science.aaw2622. 32646997 PMC7879650

[17] Choi SH, Bylykbashi E, Chatila ZK, Lee SW, Pulli B, Clemenson GD, Kim E, Rompala A, Oram MK, Asselin C, (2018). Combined adult neurogenesis and BDNF mimic exercise effects on cognition in an Alzheimer’s mouse model. Science 361, eaan8821. 10.1126/science.aan8821. 30190379 PMC6149542

[18] Miller SM, and Sahay A. (2019). Functions of adult-born neurons in hippocampal memory interference and indexing. Nat. Neurosci 22, 1565–1575. 10.1038/s41593-019-0484-2. 31477897 PMC7397477

[19] Snyder JS, and Drew MR (2020). Functional neurogenesis over the years. Behav. Brain Res 382, 112470. 10.1016/J.BBR.2020.112470. 31917241 PMC7769695

[20] Ammothumkandy A, Ravina K, Wolseley V, Tartt AN, Yu P-N, Corona L, Zhang N, Nune G, Kalayjian L, Mann JJ, (2022). Altered adult neurogenesis and gliogenesis in patients with mesial temporal lobe epilepsy. Nat. Neurosci 25, 493–503. 10.1038/s41593-022-01044-2. 35383330 PMC9097543

[21] Terreros-Roncal J, Moreno-Jiménez EP, Flor-García M, Rodríguez-Moreno CB, Trinchero MF, Cafini F, Rábano A, and Llorens-Martín M. (2021). Impact of neurodegenerative diseases on human adult hippocampal neurogenesis. Science 374, 1106–1113. 10.1126/SCIENCE.ABL5163. 34672693 PMC7613437

[22] Jokeit H, and Ebner A. (1999). Long term effects of refractory temporal lobe epilepsy on cognitive abilities: a cross sectional study. J. Neurol. Neurosurg. Psychiatry 67, 44–50. 10.1136/JNNP.67.1.44. 10369821 PMC1736446

[23] Thompson PJ, and Duncan JS (2005). Cognitive decline in severe intractable epilepsy. Epilepsia 46, 1780–1787. 10.1111/J.1528-1167.2005.00279.X. 16302858

[24] Berg AT, Zelko FA, Levy SR, and Testa FM (2012). Age at onset of epilepsy, pharmacoresistance, and cognitive outcomes: a prospective cohort study. Neurology 79, 1384–1391. 10.1212/WNL.0b013e31826c1b55. 22972641 PMC3448745

[25] Lespinet V, Bresson C, N’Kaoua B, Rougier A, and Claverie B. (2002). Effect of age of onset of temporal lobe epilepsy on the severity and the nature of preoperative memory deficits. Neuropsychologia 40, 1591–1600. 10.1016/S0028-3932(02)00012-X. 11985841

[26] Hermann B, Seidenberg M, Bell B, Rutecki P, Sheth R, Ruggles K, Wendt G, O’Leary D, and Magnotta V. (2002). The neurodevelopmental impact of childhood-onset temporal lobe epilepsy on brain structure and function. Epilepsia 43, 1062–1071. 10.1046/J.1528-1157.2002.49901.X. 12199732

[27] Smith JAD, Kirmse R, Van Enkevort E, Armacost M, Dhamija R, Trehan A, and Liu C. (2020). Improving neuropsychological seizure lateralization in Spanish-speaking people with epilepsy in the US: The need to account for education and demographic differences. Epilepsy Behav. 104, 106890. 10.1016/J.YEBEH.2019.106890. 31945663

[28] Pontón MO, Satz P, Herrera L, Ortiz F, Urrutia CP, Young R, D’Elia LF, Furst CJ, and Namerow N. (1996). Normative data stratified by age and education for the Neuropsychological Screening Battery for Hispanics (NeSBHIS): Initial report. J. Int. Neuropsychol. Soc 2, 96–104. 10.1017/S1355617700000941. 9375194

[29] Elger CE, Grunwald T, Lehnertz K, Kutas M, Helmstaedter C, Brockhaus A, Van Roost D, and Heinze HJ (1997). Human temporal lobe potentials in verbal learning and memory processes. Neuropsychologia 35, 657–667. 10.1016/S0028-3932(96)00110-8. 9153028

[30] Coras R, Siebzehnrubl FA, Pauli E, Huttner HB, Njunting M, Kobow K, Villmann C, Hahnen E, Neuhuber W, Weigel D, (2010). Low proliferation and differentiation capacities of adult hippocampal stem cells correlate with memory dysfunction in humans. Brain 133, 3359–3372. 10.1093/brain/awq215. 20719879

[31] Marín-Burgin A, Mongiat LA, Pardi MB, and Schinder AF (2012). Unique processing during a period of high excitation/inhibition balance in adult-born neurons. Science 335, 1238–1242. 10.1126/science.1214956. 22282476 PMC3385415

[32] Schmidt-Hieber C, Jonas P, and Bischofberger J. (2004). Enhanced synaptic plasticity in newly generated granule cells of the adult hippocampus. Nature 429, 184–187. 10.1038/nature02553. 15107864

[33] Toni N, and Schinder AF (2015). Maturation and Functional Integration of New Granule Cells into the Adult Hippocampus. Cold Spring Harb. Perspect. Biol 8, a018903. 10.1101/CSHPERSPECT.A018903. 26637288 PMC4691791

[34] Broadhouse KM, Mowszowski L, Duffy S, Leung I, Cross N, Valenzuela MJ, and Naismith SL (2019). Memory Performance Correlates of Hippocampal Subfield Volume in Mild Cognitive Impairment Subtype. Front. Behav. Neurosci 13, 259. 10.3389/fnbeh.2019.00259. 31849620 PMC6897308

[35] Holmes GL (2015). Cognitive impairment in epilepsy: the role of network abnormalities. Epileptic Disord. 17, 101–116. 10.1684/EPD.2015.0739. 25905906 PMC5410366

[36] Schneider-Garces NJ, Gordon BA, Brumback-Peltz CR, Shin E, Lee Y, Sutton BP, Maclin EL, Gratton G, and Fabiani M. (2010). Span, CRUNCH, and Beyond: Working Memory Capacity and the Aging Brain. J. Cogn. Neurosci 22, 655–669. 10.1162/JOCN.2009.21230. 19320550 PMC3666347

[37] Guerreiro M, Ribeiro F, and de Mendonça A. (2006). P3–039: Mild cognitive impairment: Neuropsychological performance. Alzheimers Dem. 2, S383–S384. 10.1016/J.JALZ.2006.05.1306.

[38] Mast BT, and Allaire JC (2006). Verbal Learning and Everyday Functioning in Dementia: An Application of Latent Variable Growth Curve Modeling. J. Gerontol.: B 61, P167–P173. 10.1093/GERONB/61.3.P167. 16670186

[39] Greenaway MC, Lacritz LH, Binegar D, Weiner MF, Lipton A, and Munro Cullum C. (2006). Patterns of verbal memory performance in mild cognitive impairment, Alzheimer disease, and normal aging. Cogn. Behav. Neurol 19, 79–84. 10.1097/01.WNN.0000208290.57370.A3. 16783130

[40] Pereira AC, Huddleston DE, Brickman AM, Sosunov AA, Hen R, McKhann GM, Sloan R, Gage FH, Brown TR, and Small SA (2007). An in vivo correlate of exercise-induced neurogenesis in the adult dentate gyrus. Proc. Natl. Acad. Sci. USA 104, 5638–5643. 10.1073/PNAS.0611721104. 17374720 PMC1838482

[41] Ezzati A, Katz MJ, Zammit AR, Lipton ML, Zimmerman ME, Sliwinski MJ, and Lipton RB (2016). Differential association of left and right hippocampal volumes with verbal episodic and spatial memory in older adults. Neuropsychologia 93, 380–385. 10.1016/J.NEUROPSYCHOLOGIA.2016.08.016. 27542320 PMC5154822

[42] Cai Y, Yang T, Yu X, Han X, Chen G, and Shi C. (2023). The alternate-form reliability study of six variants of the Brief Visual-Spatial Memory Test-Revised and the Hopkins Verbal Learning Test-Revised. Front. Public Health 11, 1096397. 10.3389/fpubh.2023.1096397. 37033023 PMC10073731

[43] Stark SM, Yassa MA, Lacy JW, and Stark CEL (2013). A task to assess behavioral pattern separation (BPS) in humans: Data from healthy aging and mild cognitive impairment. Neuropsychologia 51, 2442–2449. 10.1016/J.NEUROPSYCHOLOGIA.2012.12.014. 23313292 PMC3675184

[44] Baker S, Vieweg P, Gao F, Gilboa A, Wolbers T, Black SE, and Rosenbaum RS (2016). The Human Dentate Gyrus Plays a Necessary Role in Discriminating New Memories. Curr. Biol 26, 2629–2634. 10.1016/j.cub.2016.07.081. 27666968

