# Supplementary material for: Human adult neurogenesis loss corresponds with cognitive decline during epilepsy progression
Source: Cell Stem Cell. Author manuscript; Available in PMC 2026 Feb 6. (PMC12807443; doi:10.1016/j.stem.2024.11.002)
Supplement: 1 [file NIHMS2040916-supplement-1.pdf]

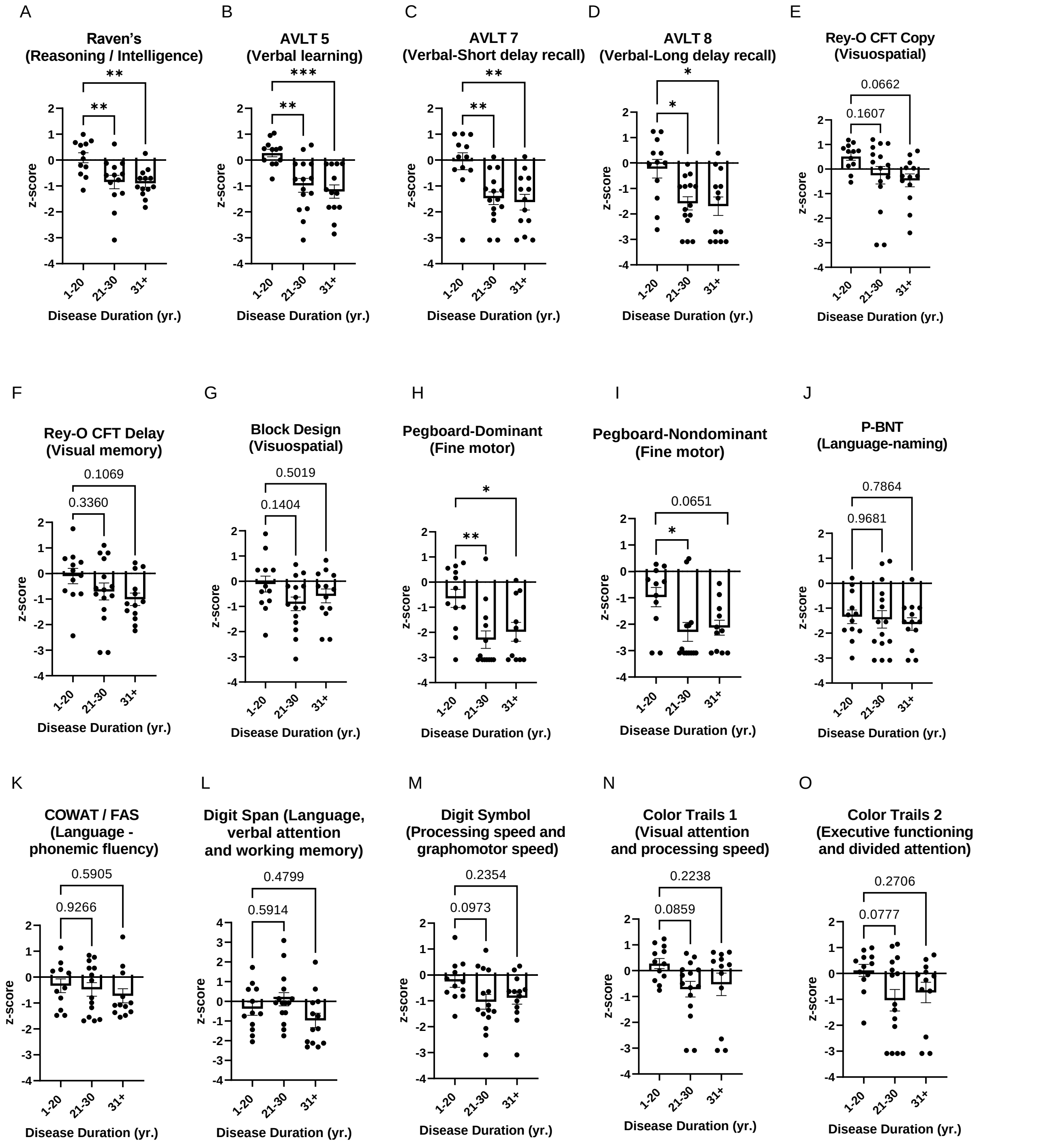

Figure S1, Related to Figure 2: Identifying critical periods of cognitive decline for 15 cognitive tests. Three DD groups were created; G1: 1-20 years DD, G2: 21-30 years DD, and G3: 31+ years DD. Cognitive z- scores between 3 groups were compared with a one-way ANOVA with post-hoc Tukey's analysis for 15 cognitive domains: A) Raven's Intelligence, B) AVLT 5 verbal learning, C) AVLT 7 verbal short-delay recall, D) AVLT 8 long-delay recall, E) Rey-O-CFT Copy visuospatial, F) Rey-O-CFT Delay visuospatial memory, G) Block Design visuospatial, H) Pegboard dominant fine motor control, I) Pegboard Non-dominant fine motor control, J) P-BNT language, K) COWAT/FAS language fluency, L) Digit Span language attention, M) Digit Symbol processing speed, N) Color trails 1 visual attention, O) Color trails 2 executive functioning. *p ≤ 0.05, ** p ≤ 0.01, *** p ≤ 0.001. All error bars represent S.E.M across individual cases.

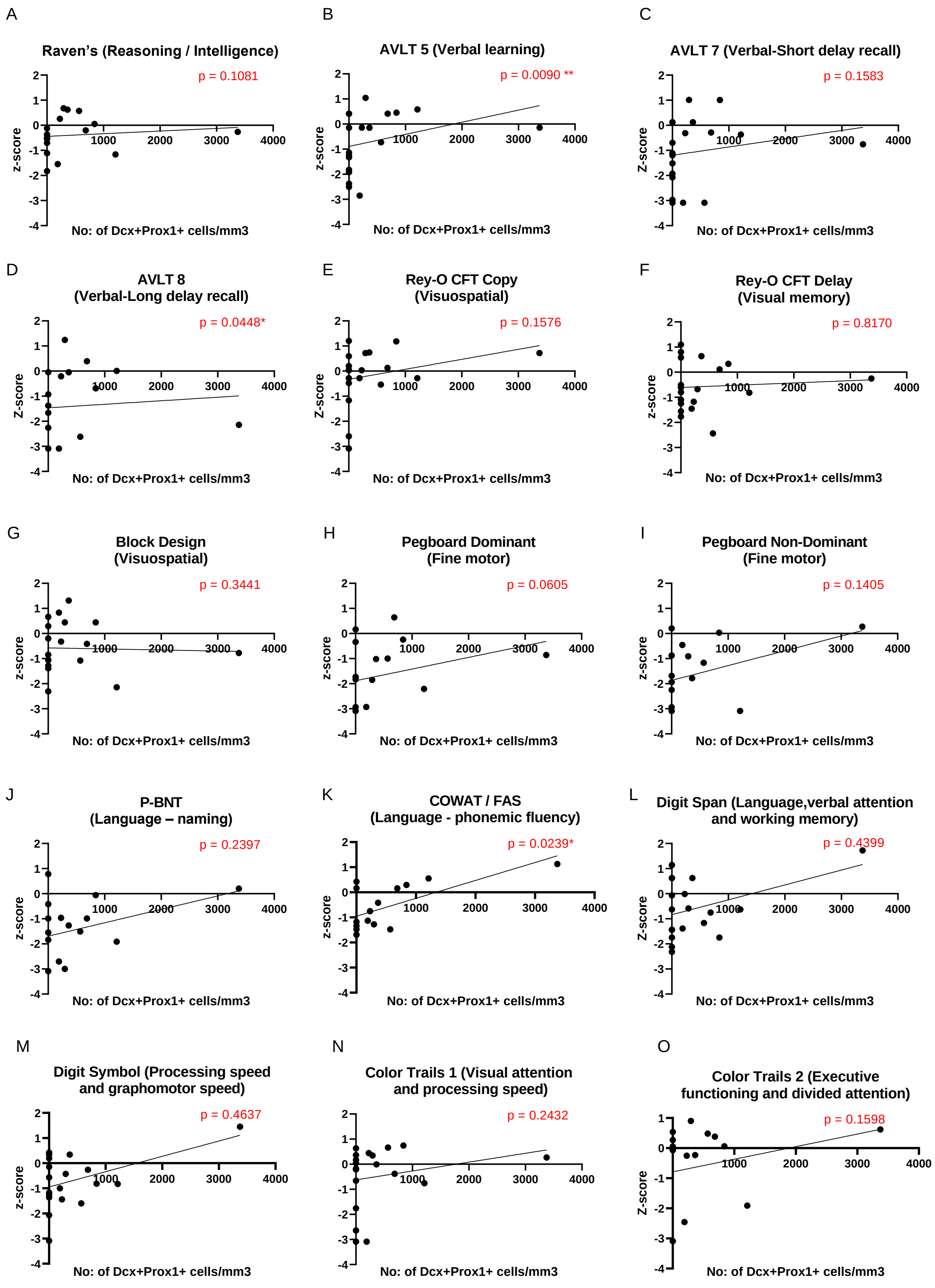

Figure S2, Related to Figure 3: Contributions of immature granule neurons to cognition.
Two-tailed Spearman's Correlation between the number of Dcx+ Prox1+ immature neurons/mm3 detected from the surgically resected hippocampus and presurgical cognitive z-scores for 15 cognitive domains: A) Raven's Intelligence, B) AVLT 5 verbal learning, C) AVLT 7 verbal short-delay recall, D) AVLT 8 long-delay recall, E) Rey-O-CFT Copy visuospatial, F) Rey-O-CFT Delay visuospatial memory, G) Block Design visuospatial, H) Pegboard dominant fine motor control, I) Pegboard Non-dominant fine motor control, J) P-BNT language, K) COWAT/FAS language fluency, L) Digit Span language attention, M) Digit Symbol processing speed, N) Color trails 1 visual attention, O) Color trails 2 executive functioning. $^{*}p \leq 0.05$, $^{**}p \leq 0.01$, $^{***}p \leq 0.001$. Multiple test adjustment for 15 cognitive tests using BH-FDR test does not yield significant q-values.

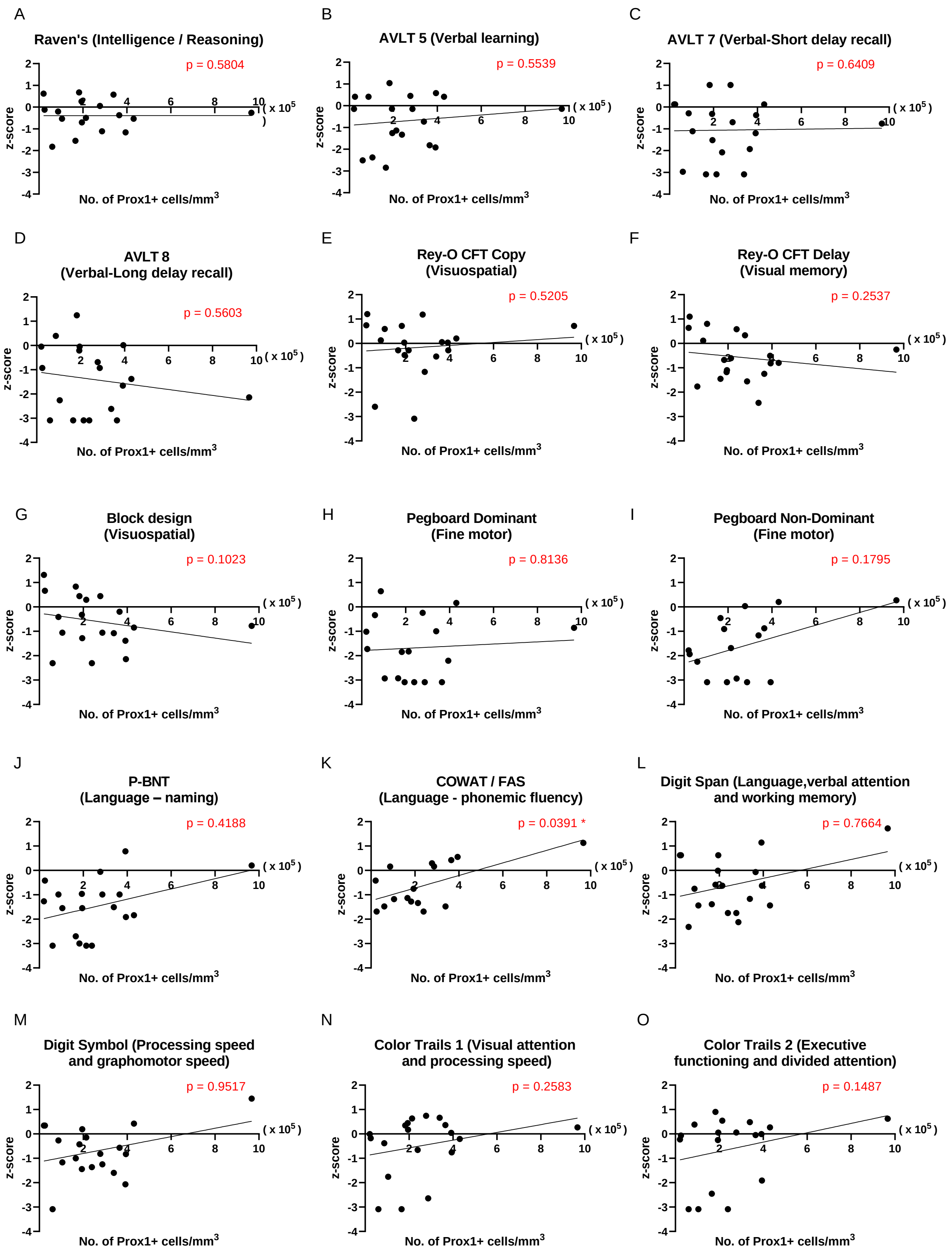

Figure S3, Related to Figure 3: Contributions of mature granule neurons to cognition.

Two-tailed Spearman's Correlation between the number of Prox1+ mature granule neurons/mm3 detected from the surgically resected hippocampus and presurgical cognitive z-scores for 15 cognitive domains: A) Raven's Intelligence, B) AVLT 5 verbal learning, C) AVLT 7 verbal short-delay recall, D) AVLT 8 long-delay recall, E) Rey-O-CFT Copy visuospatial, F) Rey-O-CFT Delay visuospatial memory, G) Block Design visuospatial, H) Pegboard dominant fine motor control, I) Pegboard Non-dominant fine motor control, J) P-BNT language, K) COWAT/FAS language fluency, L) Digit Span language attention, M) Digit Symbol processing speed, N) Color trails 1 visual attention, O) Color trails 2 executive functioning. *p ≤ 0.05, ** p ≤ 0.01, *** p ≤ 0.001. Multiple test adjustment for 15 cognitive tests using BH-FDR test does not yield significant q-values.

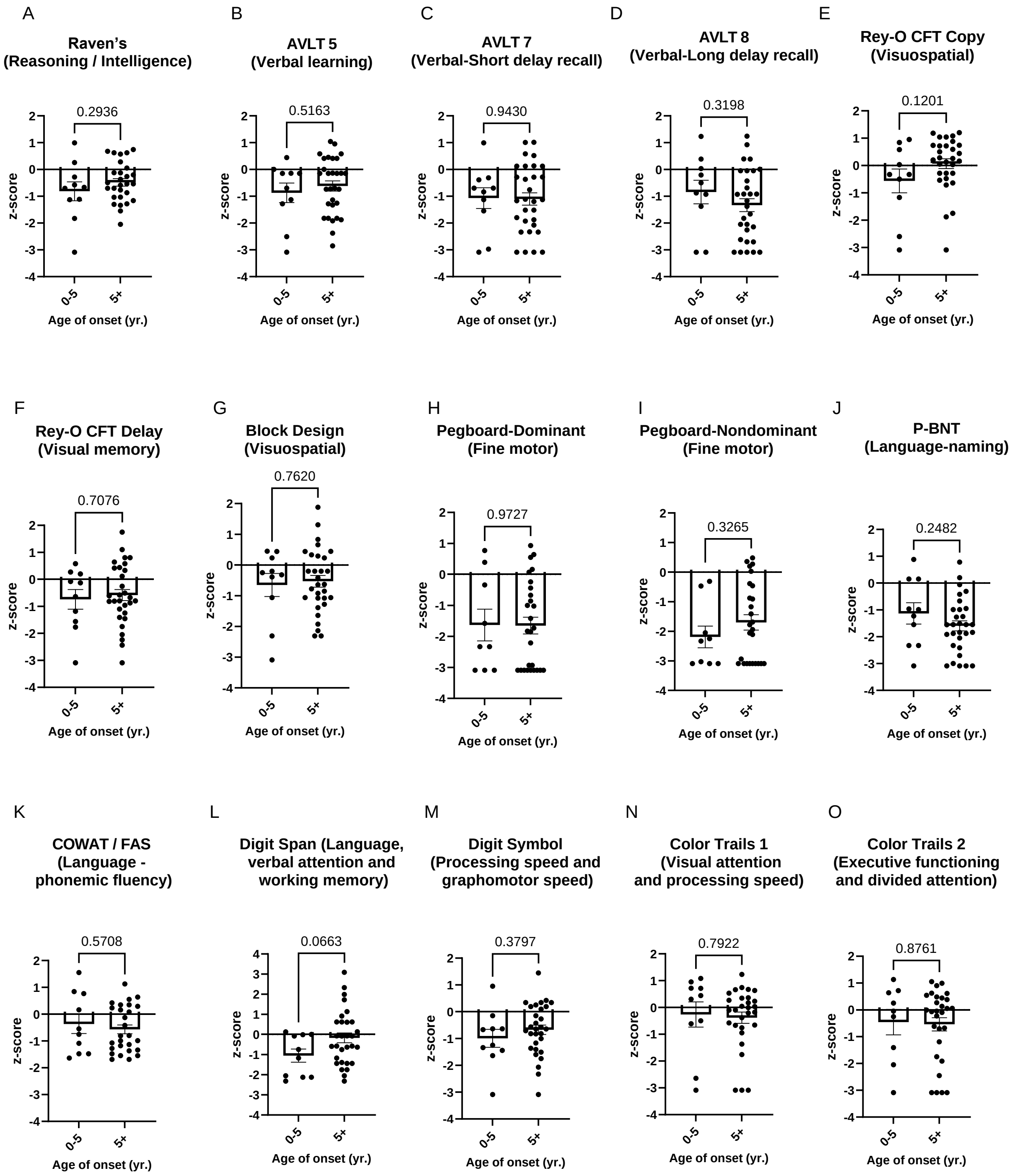

Figure S4, Related to Limitations section: Association between disease onset and cognitive decline across 15 cognitive tests. Two age of disease onset groups were created; G1: 0-5 years old, and G2: 5+ years old. Cognitive z-scores between 2 groups were compared with an Unpaired t-test for 15 cognitive domains:: A) Raven's Intelligence, B) AVLT 5 verbal learning, C) AVLT 7 verbal short-delay recall, D) AVLT 8 long-delay recall, E) Rey-O-CFT Copy visuospatial, F) Rey-O-CFT Delay visuospatial memory, G) Block Design visuospatial, H) Pegboard dominant fine motor control, I) Pegboard Non-dominant fine motor control, J) P-BNT language, K) COWAT/FAS language fluency, L) Digit Span language attention, M) Digit Symbol processing speed, N) Color trails 1 visual attention, O) Color trails 2 executive functioning. All error bars represent S.E.M across individual cases.

Subject Number	Disease duration at Neuropsychology test (yr.)	Age at Neuropsychology test (yr.)	Age of onset (yr.)	Disease Duration at surgery (yr.)	Education (yr.)	Sex	Seizure Laterality	Region of origin	Figure
MTLE 1	2	27	25	3	12	male	left	Central America	1,2,3
MTLE 2	6	21	15	10	12	female	left	Central America	1,2,3
MTLE 3	7	56	49	7	8	female	right	Mexico	1,2,3
MTLE 4	7	41	34	11	3	female	right	Mexico	1,2,3
MTLE 5	8	20	12	9	9	male	left	Mexico	1, 2
MTLE 6	8	38	30	8	12	male	left	Central America	1,2,3
MTLE 7*	8	41	33	8	12	female	right	Mexico	1,2,3
MTLE 8	15	40	25	18	6	female	left	Mexico	1,2,3
MTLE 9	16	21	5	16	9	female	left	Mexico	1, 2
MTLE 10	16	24	8	18	6	female	right	Mexico	1, 2
MTLE 11	17	27	10	19	6	male	right	Central America	1,2,3
MTLE 12	20	21	1	Not Available	11	male	right	Central America	1, 2
MTLE 13	21	38	17	21	12	female	left	Central America	1,2,3
MTLE 14	22	23	1	23	6	male	right	Central America	1, 2
MTLE 15	22	31	9	23	12	male	right	Central America	1, 2
MTLE 16	22	34	12	22	6	male	left	Central America	1, 2
MTLE 17	23	35	12	26	5	female	right	Mexico	1, 2
MTLE 18	25	37	12	26	5	female	right	Mexico	1, 2
MTLE 19	25	32	7	25	5	male	right	Mexico	1, 2
MTLE 20	26	38	12	27	6	male	right	Mexico	1,2,3
MTLE 21	26	54	28	26	14	female	right	Mexico	1,2,3
MTLE 22	27	27	0	31	12	female	left	Mexico	1, 2
MTLE 23	27	39	12	30	1	male	right	Mexico	1,2,3
MTLE 24	27	50	23	28	6	female	left	Mexico	1,2
MTLE 25	28	31	3	29	9	male	left	Mexico	1, 2
MTLE 26	28	43	15	28	7	female	left	Mexico	1, 2
MTLE 27	29	54	25	Not Available	14	female	right	Mexico	1, 2
MTLE 28	31	39	8	31	6	male	right	Mexico	1,2,3
MTLE 29	35	49	14	36	12	female	right	Mexico	1, 2
MTLE 30	38	50	12	39	3	female	right	Mexico	1, 2
MTLE 31	38	45	7	38	6	female	right	Central America	1,2,3
MTLE 32	39	54	15	39	8	female	left	Mexico	1,2,3
MTLE 33	42	48	6	42	8	female	left	Mexico	1,2,3
MTLE 34	42	48	6	43	6	female	left	Mexico	1,2
MTLE 35	43	50	7	43	6	male	left	Mexico	1, 2
MTLE 36	45	50	5	46	2	female	left	Mexico	1, 2
MTLE 37	48	52	4	Not Available	6	female	left	Central America	1, 2
MTLE 38	52	53	1	52	12	male	left	Central America	1,2,3
MTLE 39	53	56	3	53	3	female	right	Mexico	1,2,3
MTLE 40	56	57	1	56	12	male	right	Mexico	1,2,3

Table S1, Related to all figures: Clinical, demographic information and experiments performed in MTLE cases (N = 40). MTLE- Mesial Temporal Lobe Epilepsy Disease duration, Age at surgery, Age of onset and Education in years (yr.) * MTLE 7 : Highest level of immature (3373 Dcx+ Prox1 cells/mm^3) and mature granule (966850 Prox1+ cells/ mm^3) neurons

Cognitive Domain	Number of patients	Spearman's correlation coefficient (r)	p-value	q-value
**Raven's**	38	-0.5137	0.0010***	0.0075**
**Digit Symbol**	40	-0.2698	0.0923	0.1385
**Color trails 1**	39	-0.2055	0.2095	0.2857
**Color trails 2**	38	-0.2984	0.0688	0.1147
**P-BNT**	40	-0.0826	0.6125	0.6125
**COWAT/FAS**	37	-0.1435	0.3968	0.4578
**Digit Span**	40	-0.1823	0.2602	0.3253
**AVLT 5**	40	-0.5246	0.0005***	0.0075**
**AVLT 7**	40	-0.4629	0.0026**	0.0130*
**AVLT 8**	40	-0.3643	0.0208*	0.0548
**Block Design**	40	-0.1200	0.4609	0.4938
**Rey-O-CFT Copy**	40	-0.4002	0.0105*	0.0394*
**Rey-O-CFT Delay**	40	-0.3615	0.0219*	0.0548
**Pegboard dominant**	36	-0.3228	0.0548	0.1028
**Pegboard Non-dominant**	35	-0.3415	0.0447*	0.0958

Table S2, Related to Figure 1: Two-tailed Spearman's Correlation between disease duration and presurgical cognitive z-scores, followed by BH-FDR correction for 15 cognitive tests. * p, q ≤ 0.05, ** p, q ≤ 0.01, *** p, q ≤ 0.001.

Cognitive Domain	F-statistic (DFn, DFd), p value	G1, G2, G3 (M, SD)	Tukey's multiple comparison test
**Raven's**	F (2, 35) = 7.041, p= 0.0027**	G1 (M = 0.091, SD = 0.67)<br>G2 (M = -0.85, SD = 0.94)<br>G3 (M = -0.90, SD = 0.54)	G1 vs G2 0.0085**<br>G1 vs G3 0.0053**<br>G2 vs G3 0.9818
**Digit Symbol**	F (2, 37) = 2.455, p= 0.0998	G1 (M = -0.25, SD = 0.78)<br>G2 (M = -1.0 SD = 1.1)<br>G3 (M = -0.88, SD= 0.89)	G1 vs G2 0.0973<br>G1 vs G3 0.2354<br>G2 vs G3 0.9040
**Color trails 1**	F (2, 36) = 2.601, p= 0.0881	G1 (M = 0.28, SD = 0.67)<br>G2 (M = -0.72, SD = 1.2)<br>G3 (M = -0.53, SD= 1.5)	G1 vs G2 0.0859<br>G1 vs G3 0.2238<br>G2 vs G3 0.9097
**Color trails 2**	F (2, 36) = 2.618, p= 0.0868	G1 (M = 0.12, SD = 0.80)<br>G2 (M = -1.0 SD = 1.6)<br>G3 (M = -0.73, SD= 1.4)	G1 vs G2 0.0777<br>G1 vs G3 0.2706<br>G2 vs G3 0.8292
**P-BNT**	F (2, 37) = 0.2287, p= 0.7969	G1 (M = -1.3, SD = 0.95)<br>G2 (M = -1.4 SD = 1.4)<br>G3 (M = -1.6, SD = 0.92)	G1 vs G2 0.9681<br>G1 vs G3 0.7864<br>G2 vs G3 0.8938
**COWAT/FAS**	F (2, 34) = 0.5045, p =0.6082	G1 (M = -0.33, SD = 0.87)<br>G2 (M = -0.47 SD = 0.99)<br>G3 (M = -0.72, SD= 0.94)	G1 vs G2 0.9266<br>G1 vs G3 0.5905<br>G2 vs G3 0.7851
**Digit Span**	F (2, 37) = 2.507, p= 0.0953	G1 (M = -0.38, SD = 1.2)<br>G2 (M = 0.11 SD = 1.3)<br>G3 (M = -0.97, SD= 1.3)	G1 vs G2 0.5914<br>G1 vs G3 0.4799<br>G2 vs G3 0.0778
**AVLT 5**	F (2, 37) = 10.50, p = 0.0002***	G1 (M = 0.27, SD = 0.50)<br>G2 (M = -0.98, SD= 1.0)<br>G3 (M = -1.2, SD = 0.93)	G1 vs G2 0.0019**<br>G1 vs G3 0.0004***<br>G2 vs G3 0.7536
**AVLT 7**	F (2, 37) = 8.580, p= 0.0009***	G1 (M = -0.045, SD = 1.1)<br>G2 (M = -1.5 SD = 0.95)<br>G3 (M = -1.6, SD= 1.1)	G1 vs G2 0.0034**<br>G1 vs G3 0.0017**<br>G2 vs G3 0.9244
**AVLT 8**	F (2, 37) = 5.937, p= 0.0058**	G1 (M = -0.22, SD = 1.3)<br>G2 (M = -1.6 SD = 1.0)<br>G3 (M = -1.7, SD= 1.3)	G1 vs G2 0.0148*<br>G1 vs G3 0.0104*<br>G2 vs G3 0.9666
**Block Design**	F (2, 37) = 1.897, p= 0.1644	G1 (M = -0.11, SD = 1.1)<br>G2 (M = -0.90, SD = 1.0)<br>G3 (M = -0.58, SD= 0.99)	G1 vs G2 0.1404<br>G1 vs G3 0.5019<br>G2 vs G3 0.7089
**Rey-O-CFT Copy**	F (2, 37) = 2.956, p= 0.0644	G1 (M = 0.51, SD = 0.54)<br>G2 (M = -0.25, SD = 1.4)<br>G3 (M = -0.46, SD= 0.95)	G1 vs G2 0.1607<br>G1 vs G3 0.0662<br>G2 vs G3 0.8571
**Rey-O-CFT Delay**	F (2, 37) = 2.240, p= 0.1207	G1 (M = -0.10, SD = 1.0)<br>G2 (M = -0.70, SD = 1.3)<br>G3 (M = -1.0, SD= 0.87)	G1 vs G2 0.3360<br>G1 vs G3 0.1069<br>G2 vs G3 0.7414
**Pegboard dominant**	F (2, 33) = 5.956, p= 0.0062**	G1 (M = -0.65, SD = 1.2)<br>G2 (M = -2.3 SD = 1.3)<br>G3 (M = -2.0, SD= 1.2)	G1 vs G2 0.0066**<br>G1 vs G3 0.0403*<br>G2 vs G3 0.8133
**Pegboard Non-dominant**	F (2, 32) = 4.383, p= 0.0208*	G1 (M = -0.97, SD = 1.2)<br>G2 (M = -2.3 SD = 1.3)<br>G3 (M = -2.1, SD= 0.93)	G1 vs G2 0.0245*<br>G1 vs G3 0.0651<br>G2 vs G3 0.9390

Table S3, Related to Figure 2: A Critical period of cognitive impairment in MTLE patients. Three DD groups were created; G1: 1-20 years DD, G2: 21-30 years DD, and G3: 31+ years DD. Cognitive z-scores among 3 groups were compared with a one-way ANOVA with post-hoc Tukey's analysis. *p ≤ 0.05, ** p ≤ 0.01, *** p, q ≤ 0.001.

MULTIPLE TEST ADJUSTMENT FOR 4 COGNITIVE TESTS:

Cognitive Domain	Number of patients	Immature Neurons (Dcx+ Prox1+)			Mature Neurons (Prox1+)		
		Spearman's correlation coefficient (r)	p-value	q-value	Spearman's correlation coefficient (r)	p-value	q-value
**Raven's**	17	0.4039	0.1081	0.1583	-0.1435	0.5804	0.6409
**AVLT 5**	19	0.5817	0.0090**	0.0360*	0.1449	0.5539	0.6409
**AVLT 7**	19	0.3370	0.1583	0.1583	-0.1144	0.6409	0.6409
**Rey-O-CFT Copy**	19	0.3375	0.1576	0.1583	-0.1572	0.5205	0.6409

MULTIPLE TEST ADJUSTMENT FOR 15 COGNITIVE TESTS:

Cognitive Domain	Number of patients	Immature Neurons (Dcx+ Prox1+)			Mature Neurons (Prox1+)		
		Spearman's correlation coefficient (r)	p-value	q-value	Spearman's correlation coefficient (r)	p-value	q-value
**Raven's**	17	0.4039	0.1081	0.2663	-0.1435	0.5804	0.7915
**Digit Symbol**	19	0.1789	0.4637	0.4968	0.0149	0.9517	0.9517
**Color trails 1**	19	0.2814	0.2432	0.3316	0.2729	0.2583	0.6458
**Color trails 2**	18	0.3458	0.1598	0.2663	0.3547	0.1487	0.6458
**P-BNT**	19	0.2834	0.2397	0.3316	0.1970	0.4188	0.7915
**COWAT/FAS**	16	0.5688	0.0239*	0.1793	0.5243	0.0391*	0.5865
**Digit Span**	19	0.1884	0.4399	0.4968	0.0730	0.7664	0.8717
**AVLT 5**	19	0.5817	0.0090**	0.1350	0.1449	0.5539	0.7915
**AVLT 7**	19	0.3370	0.1583	0.2663	-0.1144	0.6409	0.8011
**AVLT 8**	19	0.4650	0.0448*	0.2240	-0.1426	0.5603	0.7915
**Block Design**	19	0.2297	0.3441	0.4301	-0.3863	0.1023	0.6458
**Rey-O-CFT Copy**	19	0.3375	0.1576	0.2663	-0.1572	0.5205	0.7915
**Rey-O-CFT Delay**	19	0.0569	0.8170	0.8170	-0.2754	0.2537	0.6458
**Pegboard dominant**	17	0.4673	0.0605	0.2269	-0.0617	0.8136	0.8717
**Pegboard Non-dominant**	16	0.3853	0.1405	0.2663	0.3526	0.1795	0.6458

Table S4, Related to Figure 3: Two-tailed Spearman's Correlation between presurgical cognitive z-scores and the number of 1) immature Dcx+ Prox1+ cells/mm^3 and 2) mature Prox1+ cells/mm^3 detected from the surgically resected hippocampus. Multiple test adjustments for 4 cognitive tests that showed significant decline in performance with DD (Figure 1) were performed using BH-FDR test, *p, q ≤ 0.05, ** p, q ≤ 0.01, *** p, q ≤ 0.001. Multiple test adjustment for all 15 cognitive tests using BH-FDR corrections do not yield significant q-values.
